# Extraocular muscle index as a novel indicator of inflammatory condition in graves’ ophthalmopathy patients

**DOI:** 10.3389/fendo.2025.1594828

**Published:** 2025-05-29

**Authors:** Fangkun Wu, Jin Huang, Mengdi Wang, Zhenbin Qian, Yaohua Wang, Wei Fang

**Affiliations:** National Clinical Research Center for Ocular Diseases, Eye Hospital, Wenzhou Medical University, Wenzhou, China

**Keywords:** graves’ ophthalmopathy, extraocular muscle, inflammation, indicator, crosssectional study

## Abstract

**Purpose:**

This study utilized the extraocular muscle index measured through computed tomography (CT) to objectively assess the condition of immune inflammation in patients with Graves’ ophthalmopathy.

**Methods:**

This observational study included 50 control subjects and 50 patients diagnosed with graves’ ophthalmopathy (GO). The study collected mean platelet volume (MPV) alongside other serological indicators. Additionally, thyroid function parameters, clinical activity score (CAS), and NOSPECS score were documented. In the enrolled patients, the total extraocular muscle thickness was assessed using CT at the mid-orbital region posterior to the eyeball, with measurements obtained using Image-J software. The objective of this study was to investigate the inflammatory condition by analyzing the correlation between extraocular muscle thickness and blood indices, thereby providing a reliable predictor for clinical outcomes.

**Results:**

In patients with GO, the levels of neutrophil count (NC), lymphocyte count (LC), and platelet distribution width (PDW) were significantly elevated compared to the control group (p=0.024, p=0.016, and p=0.004, respectively). Conversely, the MPV exhibited a significant decrease (p=0.005). A linear regression analysis indicated a positive correlation between MPV and extraocular muscle thickness (r=1.356, p=0.014). Notably, MPV retained a positive correlation with extraocular muscle thickness even after adjusting for potential confounding factors such as age, CAS, NOSPEC, and thyroid function.

**Conclusion:**

The extraocular muscle index serves as a reliable predictor of the overall inflammatory status in patients with GO. Consequently, we can effectively assess the systemic inflammatory status of GO patients through orbital CT, thereby providing valuable references for clinical intervention.

## Introduction

Graves’ ophthalmopathy (GO) is an autoimmune inflammatory condition frequently associated with hyperthyroidism (1). The etiology of GO is not yet fully understood. The prevailing hypothesis suggests that antigens shared between the thyroid and the orbit trigger the remodeling of extraocular muscles and orbital connective tissue through an inflammatory response (2). The clinical activity score (CAS) is the most widely used metric for assessing disease activity in patients with GO, however, it has the disadvantage of relying on subjective entirely. In addition, it is clearly a kind of bias when only using CAS scores for Asian patients (3). Consequently, there is a pressing need for an objective indicator to assess disease progression.

Mean platelet volume (MPV) measures the average size of platelets in the blood and has been linked to various diseases, including GO. Thyroid diseases, particularly hyperthyroidism and hypothyroidism, influence various hematological parameters, and changes in MPV can reflect these perturbations in thyroid function (4, 5). Patients with GO, an autoimmune condition often associated with graves’ disease, may also experience alterations in platelet dynamics due to the inflammatory nature of the disease. Previous studies have indicated that MPV correlates negatively with thyroid hormone levels, particularly free T3 and T4, suggesting that lower thyroid hormone levels may be associated with increased MPV. Increased MPV has been reported in conditions characterized by systemic inflammation, and thyroid diseases often involve inflammatory pathways that could further complicate the relationship between MPV and the prevalence of ophthalmopathy (6).

The initial quantitative study of orbital CT scans examined the common diameter and cross-sectional area of the extraocular muscle (7). The use of CT revealed that the disease severity in patients with GO is positively correlated with extraocular muscle volume and activity (8). MRI can assess active GO by comparing the signal intensity ratio of the extraocular muscles to that of normal tissue (9). However, CT has been increasingly utilized for the clinical diagnosis of GO patients due to its ease of examination and lower costs. Recently, some researchers have identified the SII as a potential parameter for evaluating disease activity and severity in GO. To our knowledge, there is a relative scarcity of clinical studies validating the inflammatory condition through orbital imaging examinations. Our study represents a pioneering effort in assessing the inflammatory condition through the measurement of extraocular muscles using CT assistance.

## Methods

### Study design and subjects

This study was conducted as a cross-sectional, observational, single-center investigation utilizing the Bartley Standard as the clinical benchmark for GO. Patients clinically diagnosed with GO at our hospital between January 2024 and September 2024 were included in the study. Additionally, 50 healthy individuals, matched for age and sex, who visited our hospital and had no systemic diseases, were also enrolled. All participants underwent a comprehensive ophthalmological examination, which included evaluations of both the anterior and posterior segments using slit-lamp techniques.

For each patient, the total thickness of the extraocular muscles was measured at the mid-orbital level using CT imaging. CAS was employed to assess the level of GO activity. Additionally, the NOSPECS score was utilized to quantify disease severity, categorized as follows: no physical signs (0); physical signs without symptoms, such as eyelid retraction (1); soft-tissue inflammation (2); proptosis exceeding 22 mm (3); extraocular muscle (EOM) involvement (4); corneal defects (5); and optic nerve compression (6). Both the CAS and NOSPECS scores were assigned by an experienced physician.

The exclusion criteria encompassed various factors, including other ocular inflammatory diseases, the use of systemic or topical medications (including hormones) within the past three months, the presence of rheumatic diseases, teratomas, active infections during the control period, anemia, and hematological disorders, as well as instances of incomplete data.

### Serology indexes and thyroid function

The study evaluated a range of hematological parameters and thyroid hormone levels. These parameters included neutrophil (NC), lymphocyte (LC), and monocyte (MC) counts to assess immune cell populations; platelet number (PLT), MPV, and platelet distribution width (PDW) to evaluate platelet-related metrics; and the neutrophil-to-lymphocyte ratio (NLR), monocyte-to-lymphocyte ratio (MLR), and platelet-to-lymphocyte ratio (PLR) as indicators of systemic inflammation and immune response. The SII, calculated as neutrophils multiplied by platelets divided by lymphocytes, was used to quantify the overall inflammatory status. Additionally, thyroid function was assessed through the measurement of free triiodothyronine (FT3), free tetraiodothyronine (FT4), and serum thyroid-stimulating hormone (TSH) to determine thyroid hormone levels, along with TSH receptor antibodies (TRAb) to evaluate the autoimmune aspects of thyroid dysfunction.

### Measurements of the extraocular muscles

**Figure 1:**
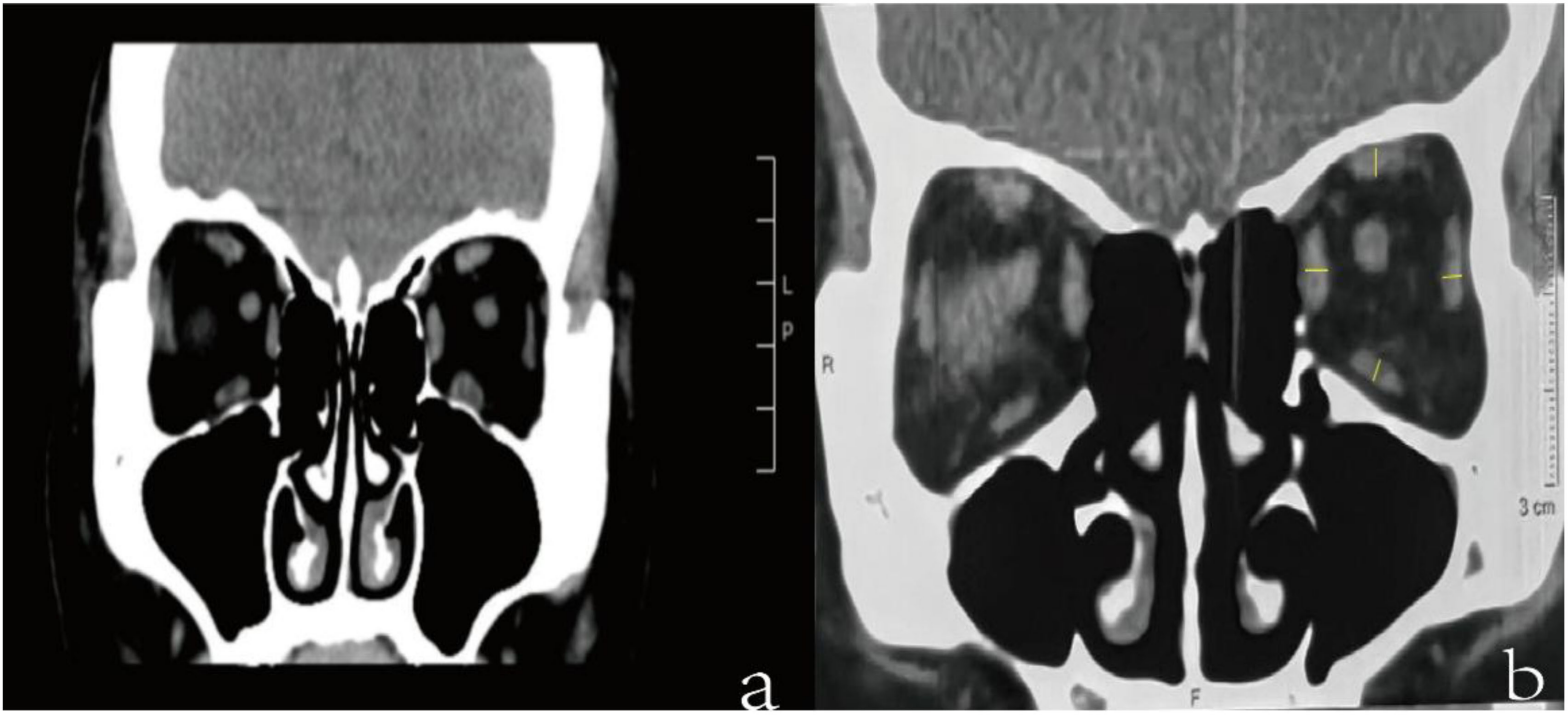
**(a)** showed the coronal section of the orbital CT scan, localized at the mid-orbital region. **(b)** referred to the measurement of extraocular muscle. We respectively measured the medial rectus, lateral rectus, inferior rectus, and superior rectus muscle and the total thickness of the extraocular muscles is obtained by summing these individual muscles.

CT was employed to assess the extraocular muscles and orbital fat tissue. The slice width was standardized at 0.625 mm, and the extraocular muscles were consistently segmented in consecutive coronal sections to ensure uniformity across all measurements. These measurements were performed on the mid-orbital slices of coronal CT images and the average was obtained after three measurements by the same measurer. The thickness of the extraocular muscle was quantified using Image-J processing software (developed by the National Institutes of Health, Bethesda, Maryland, USA). This process involved outlining the muscle profiles on coronal images and converting the grayscale to 8-bit for uniformity prior to measurement. The total EOM thickness was calculated by summing the measurements of the medial rectus, lateral rectus, inferior rectus, and superior rectus muscle. Due to the challenges in precisely distinguishing the elevator palpebrae from the superior rectus muscle, these were collectively evaluated as the superior muscle group (**Figure 1**).

### Statistical analysis

All statistical analyses were conducted using IBM SPSS version 25.0 (SPSS, Chicago, IL, USA). The normality of the data was assessed using the Shapiro-Wilk test, with parameters conforming to a normal distribution expressed as Mean ± SD. A t-test was employed to evaluate the differences in whole blood counts between the patient and control groups. Multiple linear regression analysis was utilized to examine the relationship between extraocular muscle measurements and serological indicators, age, and thyroid function indices. Correlations among the CAS, the NOSPECS classification, and whole blood counts were assessed using Spearman’s correlation analysis. Furthermore, partial correlation analysis was conducted to precisely investigate the relationship between the extraocular muscle index and blood counts, controlling for potential confounding factors. A p-value of less than 0.05 was considered statistically significant.

## Results

Complete blood counts between the GO group and the control group showed differences. The mean NC was 3.77 ± 1.72 in the GO group and 3.14 ± 0.88 in the control group (p=0.024). The mean LC was 0.40 ± 0.16 in the GO group and 0.34 ± 0.09 in the control group (p<0.05). The mean PDW was 13.45 ± 2.71 in the GO group and 12.05 ± 1.66 in the control group (p<0.01). The mean MPV was 9.79 ± 1.21 in the GO group and 10.39 ± 0.86 in the control group (p=0.005). Other serum indices showed no significant differences (**Table 1**).

**Table 1:** Comparison in whole blood counts.

Parameters	Mean ± SD		t	p
	GO(n=50)	Control Group(n=50)		
NC	3.77 ± 1.72	3.14 ± 0.88	2.313	0.024*
MC	2.20 ± 0.68	1.99 ± 0.50	1.709	0.091
LC	0.40 ± 0.16	0.34 ± 0.09	2.455	0.016*
PLT	251.52 ± 68.60	238.88 ± 52.03	1.038	0.302
PDW	13.45 ± 2.71	12.05 ± 1.66	2.989	0.004**
MPV	9.79 ± 1.21	10.39 ± 0.86	-2.851	0.005**
NLR	1.75 ± 0.79	1.63 ± 0.48	0.942	0.349
PLR	121.33 ± 36.51	126.48 ± 40.36	-0.669	0.505
MLR	0.20 ± 0.08	0.17 ± 0.04	1.517	0.134
SII	426.16 ± 211.16	394.08 ± 163.31	0.85	0.398

NC, neutrophil Count; LC, lymphocyte count; MC, monocyte count; PLT, platelet cells; PDW, platelet distribution width; MPV, mean platelet volume; NLR, neutrophil count/lymphocyte ratio; PLR, platelet cells/lymphocyte ratio; MLR, monocyte/lymphocyte ratio; SII, neutrophil count*platelet cells/lymphocyte.

* refers to p<0.05, ** refers to p<0.01.

**Figure 2:**
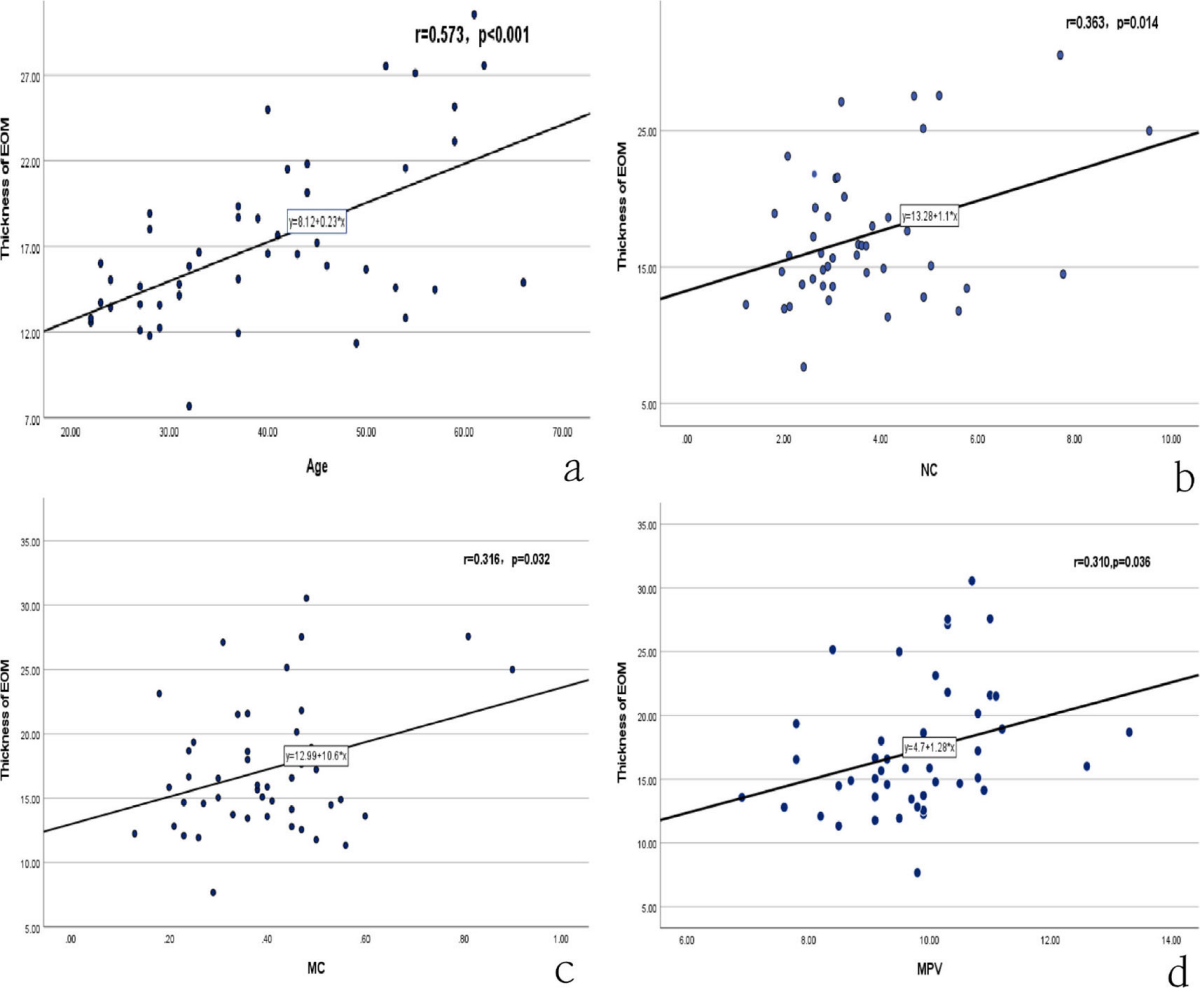
Correlation analysis between thickness EOM and age, NC, MC, MPV. NC, neutrophil count; MC, monocyte count; MPV, mean platelet volume. **(a-d)** respectively represented the correlation between thickness with age, NC, MC and MPV.

The correlation analysis showed that there was not obvious correlation between thickness of EOM with thyroid function and some blood counts (p>0.05). Worth notably, thickness of EOM was positively correlated with the age (r=0.573, p<0.01), NC (r=0.363, p<0.01), MC (r=0.647, p<0.001) and MPV (r=0.310, p<0.05) (**Figure 2**).

**Figure 3:**
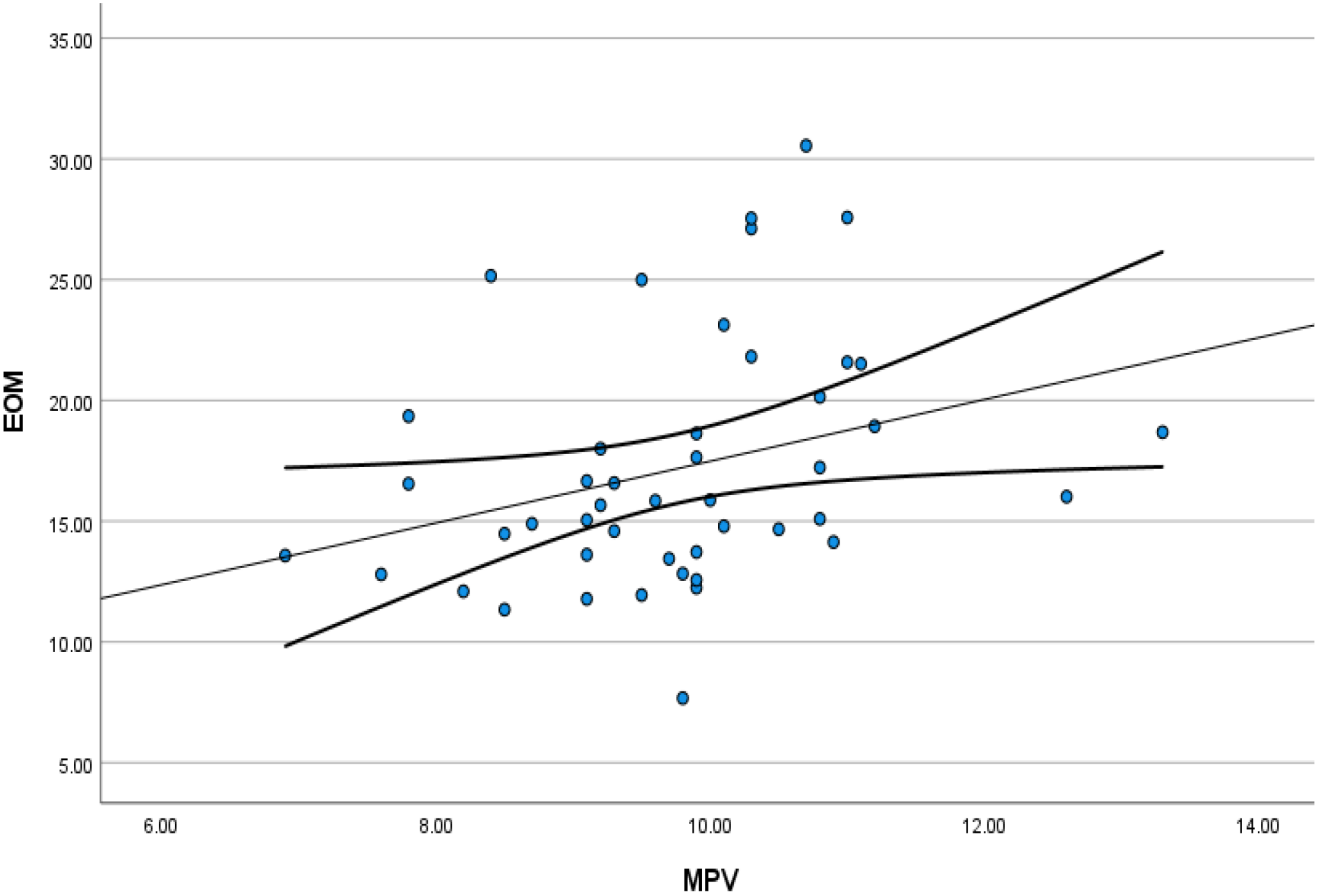
Scatter plots showing the partial correlations between the MPV and total thickness of extraocular muscle controlling for all confounding factors. MPV, mean platelet volume; EOM, total thickness of extraocular muscle.

Linear regression analysis revealed that MPV (r=1.356, p<0.05) and age (r=0.181, p<0.01) exhibited positive correlation with the thickness of extraocular muscles (**Table 2**). CAS showed no significant relationship with any blood parameters, while NOSPEC demonstrated positive correlation with NC, LC, NLR, and MLR (**Table 3**). There existed positive correlation between MPV and muscle thickness after excluding various influencing factors such as age, FT4, CAS, and NOSPEC through partial correlation analysis (r=0.494, p=0.001) (**Figure 3**).

**Table 2:** Multivariate analysis for total extraocular muscle thickness.

	OR	95% CI	p
NC	0.304	-0.837 ~ 1.445	0.605
LC	-1.321	-13.663 ~ 11.020	0.835
MPV	1.356	0.332 ~ 2.380	0.014*
Age	0.181	0.079 ~ 0.283	0.001**
FT4	0.217	-0.006 ~ 0.440	0.065

NC, neutrophil count; LC, lymphocyte count; MPV, mean platelet volume; FT4, free thyroxine; * refers to p<0.05, ** refers to p<0.01.

**Table 3:** Univariate analysis of factors associated with CAS and NOSPECS.

Parameters	CAS	NOSPECS
NC	0.175	0.432**
MC	0.186	0.129
LC	0.139	0.377**
PLT	0.121	-0.111
PDW	-0.157	0.022
MPV	0.218	0.062
NLR	-0.038	0.389**
PLR	-0.176	-0.171
MLR	0.026	0.339*
SII	0.003	0.257

NC, neutrophil count; LC, lymphocyte count; MC, monocyte count; PLT, platelet cells; PDW, platelet distribution width; MPV, mean platelet volume; NLR, neutrophil count/lymphocyte ratio; PLR, platelet cells/lymphocyte ratio; MLR, monocyte/lymphocyte ratio; SII, neutrophil count*platelet cells/lymphocyte.

* refers to p<0.05, ** refers to p<0.01.

## Discussion

The present study is dedicated to examining alterations in complete blood counts and objective parameters of extraocular muscles via CT examination. By integrating these metrics, the research aims to utilize objective measurements of EOM to reflect systemic inflammatory condition. Complete blood counts have been extensively employed in clinical research across various conditions, including cardiovascular disease, cancer, and pregnancy, and have been established as risk factors for these diseases (10, 11). However, studies examining these associations in Graves’ disease remain relatively scarce. Previous research has indicated that higher values of the monocyte to high-density lipoprotein ratio (MHR), NLR and PLR are positively correlated with disease activity in GO (12). The balance of these parameters is disrupted in the presence of thyroid disease. Our findings reveal elevated levels of NC, LC, and PDW, alongside reduced MPV in patients with GO. Significant differences in MPV, NLR, PLR, and SII were observed between GO and healthy groups. Contrary to our findings, other studies have reported that MPV is higher in patients with abnormal thyroid function (13, 14). Changes in blood cell counts may reflect alterations in overall inflammatory status. MPV serves as an indicator of platelet activation (15). Literature consistently demonstrates that MPV is a valuable marker in assessing the progression and prognosis of various pathological conditions (16). MPV tends to elevate in certain diseases, such as inflammation, tumors, cardiovascular diseases, and tuberculosis, as well as rheumatoid arthritis (17, 18). However, some researchers argue that decreased MPV may indicate increased disease activity in patients with active ulcerative colitis (19). The reduction in MPV may be attributed to inflammatory conditions leading to excessive platelet consumption, consequently resulting in decreased platelet volume (20).

Our final results indicated that the MPV decreased in patients with GO. We speculate that this may be related to the following factors: the function of the thyroid is affected, leading to changes in hormone levels that enhance the phagocytosis of the reticuloendothelial system, thereby shortening platelet lifespan and resulting in a decrease in MPV.

Previous studies have demonstrated that extraocular muscle thickening is more pronounced in older patients, which aligns with our findings (21). Imaging examinations revealed that medial rectus (MV) enlargement was the most common manifestation, with some patients exhibiting increases in both MV and other extraocular muscles such as fat volume (FV) (22). This phenomenon may be related to the pathogenesis of GO, suggesting that increased orbital MV is an early manifestation of GO, while increased FV occurs at a later stage (23). Notably, increased MV is associated with more severe GO. Our research identified a positive correlation between muscle thickness, MPV, and age. Furthermore, after controlling for confounding variables such as age, CAS, NOSPEC, thyroid function, and other factors, MPV still demonstrated a strong positive correlation with the thickness of EOM. It has been suggested that MPV varies according to the intensity of inflammation: during low-grade inflammation, larger platelets (PLTs) are released from the bone marrow; conversely, in high-grade inflammation, the consumption of large PLTs exceeds their release, resulting in a decrease in MPV (24). The activity of inflammation can be reflected in the shape of the extraocular muscles, with initial stages of the disease characterized by muscle thickening (9, 25). In our study, MPV can, to some extent, reflect the state of inflammation. Given the relationship between MPV and EOM, we propose that EOM parameters may serve as novel biomarkers for assessing systemic inflammation. Further research is warranted to uncover additional clinical correlations between MPV and GO. Our study was limited by a relatively small sample size, which may have constrained our ability to detect relationships between certain variables. Additionally, the sample may not fully represent all populations affected by GO, potentially limiting the generalizability of our findings. Our study provides new insights into assessing disease severity through the overall inflammatory condition in GO, offering a more objective measure than the CAS. Future studies should explore the deeper connections between MPV and other hematological measures and GO activity. Furthermore, long-term follow-up studies will enhance our understanding of disease progression and improve treatment decision-making. In conclusion, a positive correlation exists between MPV and EOM thickness after excluding confounding factors such as age, TF4, CAS, and NPSPEC. Therefore, we conclude that extraocular muscle thickness may potentially serve as a biomarker for inflammatory conditions in GO.

## Data Availability

The raw data supporting the conclusions of this article will be made available by the authors, without undue reservation.
