# Correction: Extraocular muscle index as a novel indicator of inflammatory condition in graves’ ophthalmopathy patients

**DOI:** 10.3389/fendo.2026.1791317

**Published:** 2026-02-05

**Authors:** Fangkun Wu, Jin Huang, Mengdi Wang, Zhenbin Qian, Yaohua Wang, Wei Fang

**Affiliations:** National Clinical Research Center for Ocular Diseases, Eye Hospital, Wenzhou Medical University, Wenzhou, China

**Keywords:** graves’ ophthalmopathy, extraocular muscle, inflammation, indicator, crosssectional study

There was a mistake in **Figure 2** as published. Panels c and d of **Figure 2** (statistical scatter plots) were duplicated, and Panel c was incorrectly cropped with an erroneous statistical analysis value. In fact, Panel c of **Figure 2** should be a separate independent statistical scatter plot.

**Figure 2:**
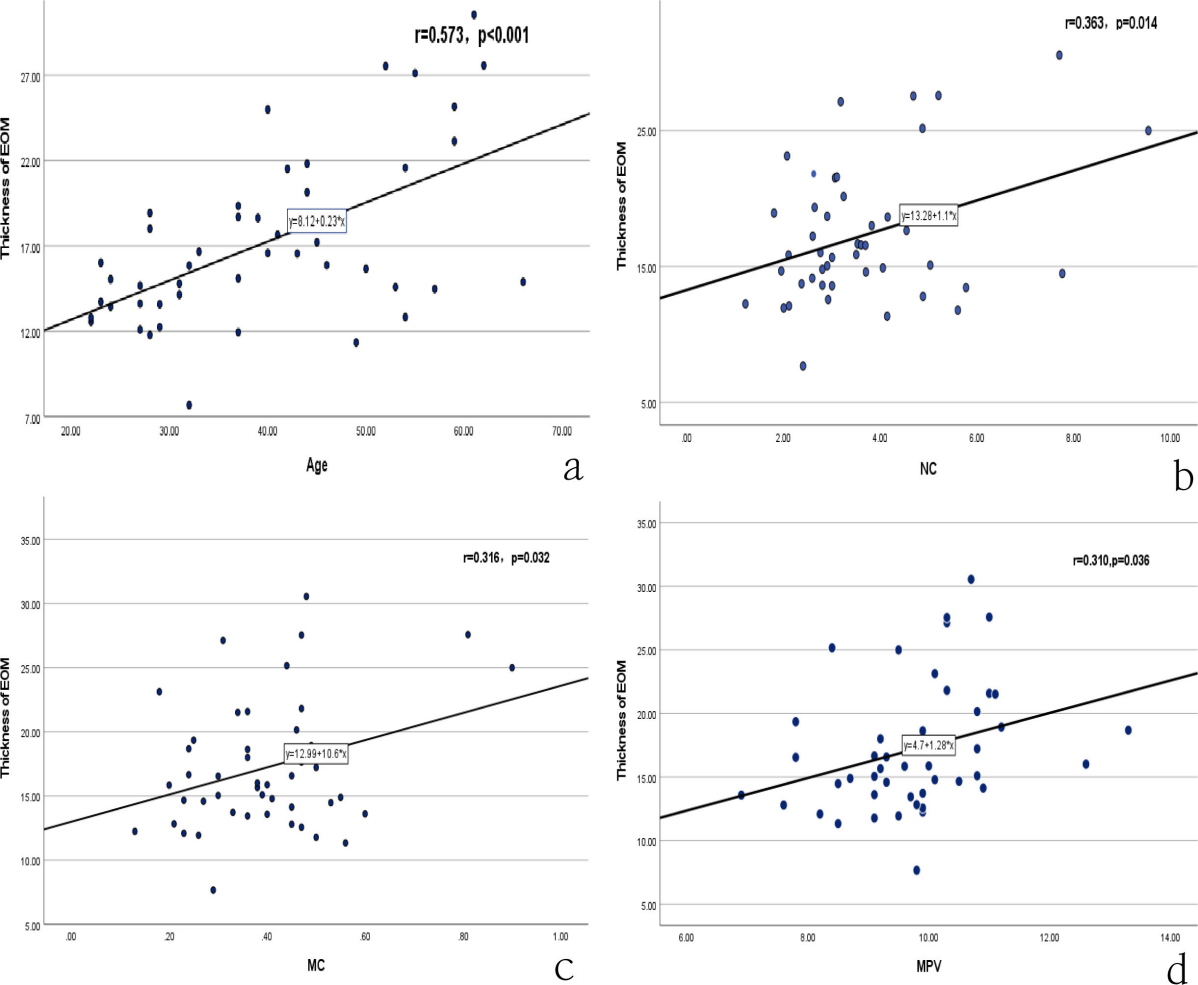
Correlation analysis between thickness EOM and age, NC, MC, MPV. NC: neutrophil count; MC: monocyte count; MPV: mean platelet volume.

The corrected **Figure 2** appears below.

In the **Introduction**, paragraph 2 was mistakenly duplicated in place of paragraph 4, and consequently omitted from the revised version.

The corrected section appears below.

**Introduction**

“Graves’ ophthalmopathy (GO) is an autoimmune inflammatory condition frequently associated with hyperthyroidism [1]. The etiology of GO is not yet fully understood. The prevailing hypothesis suggests that antigens shared between the thyroid and the orbit trigger the remodeling of extraocular muscles and orbital connective tissue through an inflammatory response [2]. The clinical activity score (CAS) is the most widely used metric for assessing disease activity in patients with GO, however, it has the disadvantage of relying on subjective entirely. In addition, it is clearly a kind of bias when only using CAS scores for Asian patients [3]. Consequently, there is a pressing need for an objective indicator to assess disease progression.

Mean platelet volume (MPV) measures the average size of platelets in the blood and has been linked to various diseases, including GO. Thyroid diseases, particularly hyperthyroidism and hypothyroidism, influence various hematological parameters, and changes in MPV can reflect these perturbations in thyroid function [4, 5]. Patients with GO, an autoimmune condition often associated with graves’ disease, may also experience alterations in platelet dynamics due to the inflammatory nature of the disease. Previous studies have indicated that MPV correlates negatively with thyroid hormone levels, particularly free T3 and T4, suggesting that lower thyroid hormone levels may be associated with increased MPV. Increased MPV has been reported in conditions characterized by systemic inflammation, and thyroid diseases often involve inflammatory pathways that could further complicate the relationship between MPV and the prevalence of ophthalmopathy [6].

The initial quantitative study of orbital computed tomography (CT) scans examined the common diameter and cross-sectional area of the extraocular muscle [7]. The use of CT revealed that the disease severity in patients with GO is positively correlated with extraocular muscle volume and activity [8]. Magnetic resonance imaging (MRI) can assess active GO by comparing the signal intensity ratio of the extraocular muscles to that of normal tissue [9]. However, CT has been increasingly utilized for the clinical diagnosis of GO patients due to its ease of examination and lower costs. Recently, some researchers have identified the SII as a potential parameter for evaluating disease activity and severity in GO. To our knowledge, there is a relative scarcity of clinical studies validating the inflammatory condition through orbital imaging examinations. Our study represents a pioneering effort in assessing the inflammatory condition through the measurement of extraocular muscles using CT assistance.”

The original version of this article has been updated.

